# Diagnostic scope in out-of-hours primary care services in eight European countries: an observational study

**DOI:** 10.1186/1471-2296-12-30

**Published:** 2011-05-13

**Authors:** Linda AMJ Huibers, Grete Moth, Gunnar T Bondevik, Janko Kersnik, Carola A Huber, Morten B Christensen, Rüdiger Leutgeb, Armando M Casado, Roy Remmen, Michel Wensing

**Affiliations:** 1 Scientific Institute for Quality of Healthcare, Radboud University Nijmegen Medical Centre, P.O. Box 9101, 114 IQ healthcare, 6500 HB Nijmegen, The Netherlands; 2 Research Unit for General Practice, University of Aarhus, Bartholins Allé 2, 8000 Aarhus C, Denmark; 3 Department of Public Health and Primary Health Care & National Centre for Emergency Primary Health Care, University of Bergen, Uni Research Kalfarveien 31, N-5018 Bergen, Norway; 4 Medical Faculty, Department of Family Medicine, University of Ljubljana, Poljanski nasip 58, SI-1000 Ljubljana, Slovenia; 5 Institute of General Practice and Health Services Research, University of Zurich, Pestalozzistrasse 24, 8091 Zurich, Switzerland; 6 Department of General Practice and Health Services Research, University Hospital Heidelberg, Voßstrasse 2, D-69115 Heidelberg, Germany; 7 Primary Health Care Research Unit, Catalan Institute of Health, C/Sant Elies 42.08006, Barcelona, Spain; 8 Department of Primary and Interdisciplinary care, University of Antwerp,Universiteitsplein 1, 2610 Wilrijk Antwerpen, Belgium

**Keywords:** primary health care, after-hours care, diagnosis

## Abstract

**Background:**

In previous years, out- of-hours primary care has been organised in large-scale organisations in many countries. This may have lowered the threshold for many patients to present health problems at nights and during the weekend. Comparisons of out-of-hours care between countries require internationally comparable figures on symptoms and diagnoses, which were not available. This study aimed to describe the symptoms and diagnoses in out-of-hours primary care services in regions in eight European countries.

**Methods:**

We conducted a retrospective observational study based on medical records from out-of-hours primary care services in Belgium, Denmark, Germany, the Netherlands, Norway, Slovenia, Spain, and Switzerland. We aimed to include data on 1000 initial contacts from up to three organisations per country. Excluded were contacts with an administrative reason. The International Classification for Primary Care (ICPC) was used to categorise symptoms and diagnoses. In two countries (Slovenia and Spain) ICD10 codes were translated into ICPC codes.

**Results:**

The age distribution of patients showed a high consistency across countries, while the percentage of males varied from 33.7% to 48.3%. The ICPC categories that were used most frequently concerned: chapter A 'general and unspecified symptoms' (mean 13.2%), chapter R 'respiratory' (mean 20.4%), chapter L 'musculoskeletal' (mean 15.0%), chapter S 'skin' (mean 12.5%), and chapter D 'digestive' (mean 11.6%). So, relatively high numbers of patients presenting with infectious diseases or acute pain related syndromes. This was largely consistent across age groups, but in some age groups chapter H ('ear problems'), chapter L ('musculoskeletal') and chapter K ('cardiovascular') were frequently used. Acute life-threatening problems had a low incidence.

**Conclusions:**

This international study suggested a highly similar diagnostic scope in out-of-hours primary care services. The incidence rates of acute life-threatening health problems were low in all countries.

## Background

An increasing number of primary care contacts take place out-of-hours, particularly since major organisational reforms in many European countries in the past years[1-6]. These organisational reforms were planned to meet various challenges facing out-of-hours care, including shortages of GPs, high workload and reduced motivation of GPs to provide out-of-hours care, and an increasing demand for out-of-hours care from patients[7-10]. In a previous study we found that many countries were confronted with these problems and that many countries have similar policies for out-of-hours care, such as the development of large-scale organisations[11]. Little is known about symptoms and diagnoses presented in out-of-hours services since these organisational reforms were implemented and the number of out-of-hours contacts increased throughout the world.

A study in Australia, New Zealand and the United States showed remarkably consistent patterns in health problems presented in primary care during office hours[12]. Musculoskeletal, cardiovascular, and ear/nose/throat problems each accounted for about 15% of the contacts in primary care. Skin, psychosocial, and respiratory problems accounted for approximately 10% of the contacts. The consistency of these patterns across countries was remarkable, because the absolute number and length of visits of patients in primary care as well as the health care systems varied substantially between these countries. This study provided crucial information for international comparisons of primary care during office hours.

However, it is unclear whether these figures can be generalised to out-of-hours primary care. In the Netherlands, patients at out-of-hours primary care settings presented with a wide range of complaints, mainly related to infections[1,13]. Most complaints presented at GP cooperatives were new and concerned acute problems[14]. Major categories of presenting complaints at out-of-hours primary care settings in the UK were digestive, respiratory, and viral/non-specific complaints. At accident and emergency (A&E) departments patients presented these complaints in smaller percentages and mostly visited with musculoskeletal problems[6,15]. Internationally comparable figures on symptoms and diagnoses in out-of-hours primary care were not yet available. Thus the aim of this study was to document and compare patients' symptoms and diagnoses in out-of-hours primary care services in regions in eight European countries.

## Methods

### Study design

We conducted an observational study based on retrospective data-collection from (computerised) medical records. The study was conducted by EurOOHnet, a European research network for out-of-hours primary health care http://www.euroohnet.eu. Participants from the following countries participated in this study: Belgium, Denmark, Germany, the Netherlands, Norway, Slovenia, Spain, and Switzerland.

### Settings

In each country, one to three regions with at least one out-of-hours primary care and emergency service were selected. Most settings were large-scale out-of-hours primary care settings (Additional file 1, *Table S1*). The exception was Switzerland, where no large-scale organisations for out-of-hours primary care existed.

In most regions, primary care providers (physicians and nurses) as well as hospital providers (physicians and nurses at A&E departments) were involved in providing out-of-hours care. In Germany the GPs were assisted by other physicians. In a number of regions the out-of-hours primary care setting delivered home visits. Different health care professionals performed telephone triage and advice such as GPs, nurses, and other health care workers. The opening hours were largely similar: all settings were open during weekends and on public holidays, although the exact opening hours varied slightly. The primary care settings were freely accessible to the general public.

### Study population

All patients who contacted an out-of-hours primary care setting from the participating regions were included in the study population. We aimed to include patient contacts in the spring of 2009. First contacts of a disease episode were included, as were repeated contacts of different episodes of the same patients. Excluded were telephone stalkers and contacts with an administrative reason for encounter (e.g. prescription).

### Procedures and variables

Data collection was the responsibility of the national researchers. We aimed to include 1000 patient contacts for each out-of-hours primary care setting. If the number of contacts exceeded 1000, a random selection procedure was performed. Measures included: gender and age of the patient (in predefined categories), patient symptoms and/or diagnosis by a professional. These symptoms and diagnoses were coded with the International Classification of Primary Care (ICPC)[16]. The ICPC allowed classification of symptom and disease diagnoses, using symptom codes and diagnosis codes. Accordingly, the term diagnosis in this paper referred to symptoms diagnoses as well as to disease diagnoses, as evaluated at the end of a contact by a professional.

ICPC coding was not routinely performed in all countries, and national researchers manually coded contacts afterwards with a symptom or disease diagnosis if necessary. Only ICD10 codes were available for the contacts of Slovenia and Spain. We recoded these ICD10 codes into ICPC codes, using a converter from Denmark http://www.dak-e.dk/icpc/ and we checked this procedure using a converter from the WHO http://apps.who.int/classifications/apps/icd/icd10online. Finally, possibly ambiguous contacts from Slovenia and Spain were discussed with national researchers. For detailed information on the final data set we refer to Table 1.

**Table 1:** Information on data collection and data sets

	Countries							
	**Belgium**	**Denmark**	**Germany**	**Netherlands**	**Norway**	**Slovenia**	**Spain**	**Switzerland**

**Contacts (N)**	1368	974	1076	2048	3000	2637	1402	649

**Period of data collection**	22-01-2010 until 22-02-2010	1-05-2009 until 30-06-2009	1-05-2009 until 30-06-2009	1-05-2009 until 30-06-2009	01-05-2009 until 30-06-2009	01-05-2009 until 30-06-2009	01-05-2009 until 10-06-2009	Jan-Febr 2009, Aug-Sept 2009

**Regions and population size**	1: North, urban only, 110000 inhabitants (N = 679) 2: North, small town & rural, 77000 inhabitants (N = 689)	North West, urban & rural, 1250000 inhabitants (N = 974)	South West, rural, 50000 inhabitants (N = 1076)	1. South East, urban, 315000 inhabitants (N = 1074) 2. Central, urban & rural, 175000 inhabitants (N = 974)	1. West, urban & rural, 25000 (N = 1000) inhabitants 2. East, urban & rural, 100000 inhabitants (N = 1000) 3. East, urban & rural 63000 inhabitants (N = 1000)	1. North West, urban & rural, 30000 inhabitants (N = 985) 2. Central, urban, 250000 inhabitants (N = 684) 3. East, urban & rural, 30000 in habitants (N = 968)	1 and 2. North East, urban, 160000 + 136000 inhabitants (N = 1402)	North, urban, 400000 inhabitants (N = 649)

**Inclusion**	All patients	Computerised randomisation	Face to face and telephone contacts	According to criteria; computerised randomisation	Computerised randomisation	Only face to face contacts; computerised randomisation for region 1 and 3	According to criteria; computerised randomisation	In 1^st ^and 2^nd ^period, all patient contacts were counted but further patient info was only documented for 1^st ^, 2^nd ^and last contact

**Exclusion**	Not applicable	Not applicable	Not applicable	Prescriptions; follow-up contacts	Not applicable	Telephone contacts (not systematically recorded); prescriptions	Not applicable	Almost all

**Missings**	No missings	Missings for diagnosis replaced by symptoms	ICPC code is not necessary for patients with private insurance; missings for diagnosis replaced by symptoms	Missings for diagnosis replaced by symptoms	No missings	No missings	No missings	Gender: this variable was not on the questionnaire in the 2^nd ^period; Age: compliance of GP's filling out questionnaire

**ICPC**	ICPC2	ICPC	ICPC, several codes per contact	ICPC 1, partly manual	ICPC 2, several codes per contact	ICD10, manually recoded into ICPC2	ICD10, manually recoded into ICPC2	ICPC, several codes per contact

**Recording symptoms/diagnosis**	By GP	By GP	Symptoms by nurse and GP; diagnosis by GP	Symptoms by triage nurse (telephone consult) and additional by GP; diagnosis by GP	Symptoms by triage nurse (telephone consult) and additional by GP; diagnosis by GP and sometimes by a nurse in telephone consultations	By GP	By GP	By GP

**Coding by**	GP, an administrator, reviews all reports (RFE recoded by GP of group)	Coding afterwards by a medical student	Nurses coded symptoms at the beginning. GP/physician coded diagnosis at the end	GP at the end of the contact; a trained nurse filled in the blanks	GP at the end of the contact; sometimes by a nurse at the end of a telephone consultation	Manual by researcher from the Netherlands; checked by national researcher	Manual by researcher from the Netherlands; checked by national researcher	Two researchers (MD)

Countries had different health care services; therefore opening hours of out-of-hours services varied across countries.

Swiss: patients contacting via the EMST (triage telephone line) were included and data collection was performed with a paper survey.

### Data analysis

Patient contacts were the unit of analysis. As ICPC codes of professional diagnosis were present in all data sets and codes of patients' symptoms were frequently missing, we further analysed the codes of the diagnosis. Some countries had more than one ICPC code per contact. We added up all codes present in these data sets and calculated percentages using the total number of codes used (not the total number of contacts). For the Netherlands, Germany and Denmark, we observed respectively 20.6%, 27.6% and 8.5% missings in ICPC diagnosis. We therefore substituted these with ICPC symptom codes where possible. We stratified the main ICPC chapters for different age groups, because we expected diagnosis codes to be related to the age of patients.

## Results

### Patient characteristics

In all out-of-hours primary care settings less than half of the contacts concerned male patients, with percentages varying from 33.7% in Germany to 48.3% in Denmark (Table 2). The mean age differed, from 34.1 years in Denmark up to 58.9 years in Switzerland.

**Table 2:** Patient characteristics and age in categories (%)

	Countries							
	**Belgium** **(N = 1368)**	**Denmark** **(N = 974)**	**Germany** **(N = 1076)**	**Netherlands** **(N = 2048)**	**Norway** **(N = 3000)**	**Slovenia°** **(N = 2637)**	**Spain** **(N = 1402)**	Switzerland* (N = 649)

**Gender **(male; %)	44.3	48.3	33.7	47.5	46.7	46.9	43.3	36.5
**Age **(mean; year)	36.2	34.1	42.5	35.2	35.4	51.2*	n.a.	58.9

								

**Age in categories (years)**								

0-4	15.0	19.6	9.1	13.4	15.9	5.6	4.7	0
5-11	9.4	8.1	9.9	10.2	9.7	5.4	7.6	0.5
12-17	4.6	5.6	6.9	7.0	5.3	2.8	5.6	1.4
18-24	8.2	11.4	8.6	9.9	10.1	7.9	12.3	5.1
25-34	15.1	11.6	7.1	13.2	12.3	14.6	21.5	13.5
35-44	12.4	9.3	10.5	13.0	12.3	13.6	17.1	14.2
45-54	10.4	8.8	12.8	9.1	7.9	15.2	9.2	10.3
55-64	7.1	8.4	7.7	8.1	9.4	13.3	8.3	9.0
65-74	5.4	5.9	10.6	6.4	5.6	10.6	7.3	10.0
75 and more	12.4	11.2	16.8	9.8	11.5	11.0	6.3	36.1

* Missings: Switzerland: gender 40.1% and age 8.8% (data collection error); °Slovenia: age missing for two 2 settings (74.1%); Spain no information on age.

The age distribution of patients per country showed a high consistency (Table 2). In general, there was a peak in the young age categories, followed by a decrease until the age group 18-24 years. In the next age groups, there was an increase again that slowly declined, except for the eldest age category, which showed another peak. The age distributions in Belgium, Denmark, the Netherlands and Norway were quite comparable. Evidently, differences were observed as well. The German and Swiss samples had fewer young patients and more elderly (over 74 years of age). In Slovenia, there were few children and for Spain the peak was between 18 and 44 years.

### Diagnoses per country: ICPC chapters

Table 3 presents the distribution of diagnoses by ICPC chapter. In general, in Denmark, Norway, and to some extent the Netherlands, we found very similar health problems presented during out-of-hours. Also, Germany and Switzerland were quite similar. The ICPC codes that were used most often were from the chapters 'general and unspecified'(A), 'respiratory'(R), and 'musculoskeletal' (L). Furthermore, 'skin' (S), and 'digestive' (D) chapters were frequently used. The chapter S contained codes for wounds and bruises. In Germany and Switzerland cardiovascular diseases were similarly coded (respectively 10.8% and 9.2%).

**Table 3:** Incidence in different ICPC chapters (% of contacts per country)

	Belgium (N = 1368)	Denmark (N = 974)	Germany (N = 1076)	Netherlands (N = 2048)	Norway (N = 3000)	Slovenia (N = 2637)	Spain (N = 1402)	Switzerland (N = 649)
**A. General and unspecified**	**11.6**	**19.1**	**13.4**	**15.3**	**17.8**	7.2	7.6	**13.7**
**B. Blood, blood forming organs and immune mechanism**	0.4	0.0	0.7	0.2	0.4	0.4	0.1	0
**D. Digestive**	**21.1**	11.3	6.6	9.4	9.7	**11.3**	**10.0**	**13.7**
**F. Eye**	2.2	3.3	3.6	4.6	4.6	6.2	4.2	0.7
**H. Ear**	6.0	3.8	4.6	2.5	2.3	3.0	6.4	3.4
**K. Cardiovascular**	3.7	3.0	10.8	3.5	3.1	7.7	0.4	9.2
**L. Musculoskeletal**	**10.3**	**14.5**	**11.7**	**23.3**	**14.1**	**19.8**	**12.3**	**13.9**
**N. Neurological**	2.0	3.6	2.9	3.2	3.7	4.1	2.0	3.4
**P. Psychological**	1.2	3.4	2.2	1.9	5.9	3.1	3.2	6.4
**R. Respiratory**	**27.5**	**15.7**	**18.7**	**10.8**	**14.8**	**14.6**	**38.5**	**22.4**
**S. Skin**	7.7	**12.7**	**15.5**	**18.6**	**13.9**	**15.1**	**11.3**	4.9
**T. Endocrine/metabolic and nutritional**	0.4	0.7	3.4	0.6	1.4	0.5	0.2	1.2
**U. Urological**	4.1	4.2	5.1	4.3	5.3	4.7	3.0	5.3
**W. Pregnancy, childbearing, family planning**	0.2	2.1	0	0.8	1.2	1.0	0.0	0.3
**X. Female genital**	0.7	1.7	0.3	0.6	0.9	0.6	0.5	0.4
**Y. Male genital**	0.8	0.8	0.4	0.3	0.6	0.3	0.3	0.4
**Z. Social problems**	0.1	0.2	0	0	0.3	0	0	0.7

Top 4 in bold; Percentages calculated with the total number of ICPC codes per country (see methods section)

### Diagnoses per country: ICPC chapters per age group

We also looked at the top five of ICPC chapters for the four main age categories (Additional file 1, Table S2). Again, the results were quite similar. Chapters 'general and unspecified' (A) and 'respiratory' (R) were in the top 5 for most countries and age categories, as were 'digestive' (D) and 'skin' (S). In the age group 0-17 years 'respiratory' (R) and 'general and unspecified' (A) were frequently present, as well as diagnosis related to ear problems (chapter H), although less frequently. In the middle age group (18-44 years) codes for 'musculoskeletal' (L), 'respiratory' (R), and 'skin' (S) problems were present in higher numbers. In the age group 65 years and older 'cardiovascular' diagnosis (K) entered the top five of ICPC chapters.

Additional file 1, table S3 shows the top 10 of ICPC codes used in each country. Again, we found consistency across countries. ICPC codes present in three or more countries included: acute upper respiratory infection (R74), cystitis/other urinary infection (U71), laceration/cut (S18), tonsillitis acuta (R76), acute bronchitis/bronchiolitis (R78), fever (A03), abdominal pain/cramps general (D01), infectious conjunctivitis (F70), gastroenteritis (D73), musculoskeletal injury (L81), and uncomplicated hypertension (K86). Belgium, Denmark, Norway, and the Netherlands had a largely comparable top ten. Finally, we focused on a set of ICPC codes related to emergency cases, occurring in out-of-hours primary care services. Life-threatening health problems such as acute myocardial infarction (K75) and cerebrovascular accident (K90) had low incidence figures (respectively 0 to 0.8% and 0 to 1.0%). The incidence ranges for transient cerebral ischemia (K89), acute alterations of conscience (A07, N07, N88) and severe infections like pneumonia (R81), appendicitis (D88), and pyelonefritis (U70) varied from 0% up to 2.2% for pneumonia. Overall, about one in 20 patient contacts dealt with potentially life-threatening problems.

## Discussion

### Main findings

Our study found a highly similar diagnostic scope in out-of-hours primary care services across different regions in eight European countries. Particularly regions of Denmark, the Netherlands, and Norway showed a high consistency. Acute life-threatening health problems had a low incidence in all regions. We found relatively high numbers of patients in out-of-hours primary care present with infectious diseases, such as respiratory and viral infections, or with acute pain related syndromes. Corresponding diagnosis codes were mainly from respiratory, musculoskeletal, skin, and digestive chapters. We found some differences between the regions, for instance related to the distribution of patient age and frequency of coding from chapter A ('general and unspecified').

### Interpretation

The consistency of the diagnostic scope across regions was also found in primary care within office hours[12]. Interpretation of the findings was challenging, due to possible effects of differences in the coding process, characteristics of patient population, the health care system, and the study method used. Nevertheless, some general trends could be observed.

Regarding patient characteristics, our results were consistent with previous studies. More women had contacted out-of-hours primary care than men, who tended to visit A&E departments[1,17,18]. A high proportion of children attended the out-of-hours settings in most countries, a finding that was also observed in earlier studies[1,18]. This could be the reason for the large number of contacts for infectious problems, which are highly prevalent in children. The relatively identical age distribution and ICPC codes of patients from Denmark, the Netherlands, and Norway suggests the similarity of the out-of-hours health care organisation as well as the role of the GP as a gate keeper[11,9,19]. Likewise the differences found between some regions of countries may be explained by variations in the health care organisation across Europe. For example, in Slovenia out-of-hours care for children in one of the observed settings is performed by primary care pediatricians,[20] whereas in Denmark, Norway, and the Netherlands these patients frequently visit GP cooperatives. Organisation of out-of-hours (primary) care and the role of primary care in general can be linked with patients' reasons for encounter, and subsequently diagnosis. If other organisational settings exist and are accessible out-of-hours (e.g. A&E department, specialists), this may influence the flow of patients. Stratifying the ranking of ICPC chapters for age showed that differences between regions could at least partly be explained by this variation in age distribution.

A consistent finding across countries was that the large majority of patients presented at primary out-of-hours care settings with non-acute, non-life-threatening health problems. Data on urgency assessment, which were available for six countries, supported this impression. Research on self-referring patients at A&E departments and GP cooperatives has shown similar results[1]. While previous research has found that some urgent health problems are overlooked in out-of-hours care[21]. We suggest that the large majority of patients' symptoms and diagnoses are not life-threatening and not urgent from a medical perspective. On the other hand, patients may perceive the presented health problem as urgent, potentially urgent (e.g. they feel incompetent to assess this), or urgent because of non-medical reasons (e.g. lack of time during office-hours)[17]. Despite the low incidence of life-threatening health problems, professionals in out-of-hours primary care should remain alert.

The high frequency of chapter A codes ('general and unspecified') might partly be related to a lack of specific coding by health care professionals. Furthermore, this could reflect an early stage of presented acute symptoms, such as A03 ('fever'), A77 ('viral disease other'), and non-diseases as death (A96). Also, it might be inherent to primary care, which has a higher probability of nonspecific complaints and diagnosis. Other chapters used frequently were 'musculoskeletal' and 'skin', which both contain injury related codes, such as wounds and bruises. These are one of the main reasons for seeking out-of-hours health care[14].

### Limitations

Some limitations of the study should be mentioned. Our aim was to include data of similar periods for all regions, in order to avoid seasonal effects. The contacts of Belgium and Switzerland occurred during the winter period, a fact that might have influenced the frequency of health problems presented, such as respiratory infection and fever. Furthermore, we included one to three regions per country. In some countries a regional variation in out-of-hours primary care organisation and population characteristics may be observed. Therefore, the selected region(s) might not be representative for the whole country. So, our comparison partly is of regions of eight different countries.

Some differences in the coding process had to be accepted, such as numbers of codes used per contact and per setting, individual coding decision in a particular case (such as choice for diagnosis codes instead of symptoms codes) and relation of coding with practice income. This might have influenced the content of the tables to some extent, but it is difficult to predict in what direction. In some regions ICPC were deduced from ICD10 codes, which may have induced information bias. In case of retrospective coding, the quality of the coding depended on the quality of the medical record of the out-of-hours service. The risk of information bias due to coding differences was reduced by clustering our main results in more general categories of the ICPC chapters. Data from the Netherlands showed similar patterns as in earlier research,[1] which suggests that the methods were valid. We primarily focused on professional diagnosis codes, but for three regions we substituted missings on professional diagnosis codes with patients' symptoms codes. This allowed us to keep as close as possible to the original data without exclusion, although this might have introduced some information bias. Often, the diagnosis as reported by the professional is from the same ICPC chapter as the patients' symptom. Also, coding of the professional diagnosis can be with a symptom code or a disease diagnosis code.

### Implications

The similarity of diagnostic scope at out-of-hours primary care is important for comparisons of out-of-hours care across countries. Moreover, it stimulates international collaboration in clinical studies in this setting. For instance, studies on the use of antibiotics are warranted given the increasing numbers of resistant bacteria and the relation to antibiotics use[22]. In our study, the ICPC code U71 ('cystitis/urinary infection other') was frequently used and this subgroup could be analysed in epidemiologic cross national research focusing on actual clinical behaviour and the prescription of antibiotics[23,24].

The high proportion of non-life-threatening health problems presented poses serious questions for policy makers, particularly in a time of economic challenges, an ageing population, and expected shortages of health care professionals. The trend towards larger organisations for out-of-hours care is unlikely to be reversed, but managing the increasing patient demand is a crucial challenge. A previous cross national survey showed that a large diversity of organisational models for out-of-hours care exists[11]. International studies can provide relevant information for policy makers in the ongoing discussion and the reforming of the organisation of out-of-hours primary care.

## Conclusions

The organisation of out-of-hours primary care has changed in many European countries, with an overall trend towards large-scale organisations. Comparisons across countries require knowledge of patients' symptoms and diagnoses, but internationally comparable figures on the diagnostic scope in this setting were not available. This study in regions of eight European countries found a highly similar diagnostic scope in out-of-hours primary care services. Patients presented relatively often with infectious diseases, such as respiratory and viral infections, or with acute pain related syndromes. Also, the incidence rates of acute life-threatening health problems were low in all countries. Our results imply the possibilities for international multi-centre studies in this setting that can provide relevant information for policy makers. Also, the low incidence rates of acute life-threatening health problems highlight the challenge for professionals to detect these cases.

## Ethical approval

Participants from the countries sought contact according to national or regional regulations.

For Denmark, Norway, Slovenia and Spain, according to national regulations, research based on non-person-identifiable registry data is not to be notified to the regional/national ethic committee.

For the Netherlands, the Arnhem-Nijmegen ethical committee waived approval for this study.

For Belgium and Switzerland, approval of the study was given by the local ethics committee.

For Germany, the data originated from the CONTENT project. The study protocol of CONTENT was approved by the ethics committee of the University of Heidelberg (approval number 442/2005).

## Competing interests

The authors declare that they have no competing interests.

## Authors' contributions

LH contributed to the design of the study, coordinated the data collection, analysed and interpreted the data and drafted the manuscript. GM coordinated national data collection, interpreted the data and drafted the manuscript. GB, JK, MC, RL, CH, AC and RR coordinated national data collection and critically read the manuscript. MW contributed to the design of the study, the interpretation of the data and the writing of the article. All authors have read and approved the final version of the manuscript.

## Funding

This study had no external funding source.

## Pre-publication history

The pre-publication history for this paper can be accessed here:

http://www.biomedcentral.com/1471-2296/12/30/prepub

## Supplementary Material

Additional file 1 **Table S1**. Out-of-hours primary care settings **Table S2**. Main ICPC chapters for diagnosis: Top 5 per age category (%) **Table S3**. Top 10 diagnosis ICPC (code and %) per country Click here for file
