# Supplementary material for: Diagnostic scope in out-of-hours primary care services in eight European countries: an observational study
Source: BMC Fam Pract. 2011 May 13;12:30. doi: 10.1186/1471-2296-12-30 (PMC3114765; doi:10.1186/1471-2296-12-30)
Supplement: Additional file 1 — Table S1. Out-of-hours primary care settings Table S2. Main ICPC chapters for diagnosis: Top 5 per age category (%) Table S3. Top 10 diagnosis ICPC (code and %) per country [file 1471-2296-12-30-S1.DOC]

| **Table S1.** Out-of-hours primary care settings | | | | | | | | |
| --- | --- | --- | --- | --- | --- | --- | --- | --- |
|  | **Countries** | | | | | | | |
|  | **Belgium** | **Denmark** | **Germany** | **Netherlands** | **Norway** | **Slovenia** | **Spain** | **Switzerland** |
| **Settings** | GP out-of-hours centre (only weekend and official holidays) | GP cooperative | Out-of-hours care centre (with GP on duty) | GP cooperative | Emergency primary health care clinic | Primary care walk-in centre | Primary care centre | GP on duty (rota group) & ‘night physician’ |
| **Opening hours**  *- Weekend & official holidays (Friday – Monday)*  *-Weekdays* | Fri 8pm- Mon 8am  Not open | Fri 4 pm- Mon 8 am  4pm - 8am | Fri 6 pm – Mon 7 am  Not open | Fri 5 pm – Mon 8 am  5 pm – 8 am | Fri 4 pm – Mon 8 am  4 pm - 8 am  Telephone advice by nurses 24/7 | 24/7  24/7 | Fri 8 pm - Mon 8 am  8 pm - 8 am | 7 am to 7 am next day GP on duty; 10 pm – 7 am (night physician, back up by GP on duty)  7 am to 7 am next day GP on duty; 10 pm – 7 am (night physician, back up by GP on duty) |
| **Telephone triage** | Not applicable | GP | Nurses | Community nurses using protocols and with back up of GPs | Nurses | In the capital direct walk-in access for trauma patients and for small children | Emergency Medical Service Telephone | Health care workers |
| **Other health care providers in the region** | Hospital | A&E department | Hospital | A&E department | Hospital (up to 13 km from the clinic) | Hospital | Hospital, Emergency Medical Service Telephone | Walk-in centres, A&E departments, Emergency Medical Service Telephone |
| **Free access** | Direct access to hospital & specialists; no gatekeeper role for GP | All consultations are preceded by a telephone call to the OOH centre | Yes; but the patients have to pay 10€ additional | Yes | Free only for children up to 16 years | Yes; direct access to hospital & specialists (only in the capital) | Yes, for tourist payment | Yes |
| **Demand** (number of contacts /1000/ year with primary care out-of hours settings) | 135 (estimation of contacts) | 535 (national figure in 2008) | 118 (regional figure in 2009) | 260 (national figure in 2009); 270 and 287 (regional figures in 2009) | 302 (national figure in 2007; telephone consultations not included) | 141 to 414 (regional figures in 2010 | 55 (regional figure in 2009) | 120 (national figure in 2002) |
| *Some countries were able to include more than one region (and/or settings). In all cases these regions (and settings) were comparable and representative, so the results were combined.* | | | | | | | | |

| **Table S2.** Main ICPC chapters for diagnosis: top 5 per age category (%) | | | | | | | | |
| --- | --- | --- | --- | --- | --- | --- | --- | --- |
| **Age in categories (years)** | **Belgium**  **(N=1368)** | **Denmark**  **(N=974)** | **Germany**  **(N=1076)** | **Netherlands**  **(N=2048)** | **Norway**  **(N=3000)** | **Slovenia**  **(N=2637)** | **Spain**  **(N=1402)** | **Switzerland**  **(N=649)** |
| 0-17 | R (35.8)  D (21.1)  A (13.2)  H (12.9)  S (8.1) | A (23.0)  S (18.0)  R (17.0)  L (11.8)  D (10.8) | R (27.4)  S (22.6)  A (15.4)  F (9.2)  H (8.5) | L (24.4)  S (20.4)  A (19.6)  R (13.6)  D (8.0) | A (24.0)  R (20.2)  S (16.7)  L (12.0)  D (8.8) | R (30.2)  S (19.2)  A (14.6)  D (11.8)  L (8.5) | R (44.0)  A (11.1)  H (11.1)  S (10.7)  L (8.7) | D (54.5)*  R (18.2)  H (9.1)  S (9.1)  U (9.1) |
| 18-44 | D (26.2)  R (25.2)  L (11.5)  A (9.3)  S (8.0) | A (17.9)  L (16.6)  R (15.6)  D (12.0)  S (11.3) | R (24.5)  L (14.0)  S (12.2)  A (11.1)  U (7.6) | L (25.4)  S (21.1)  D (11.6)  R (9.9)  A (8.1) | A (15.5)  L (14.2)  R (13.6)  S (13.6)  D (9.7) | S (17.1)  L (19.0)  R (13.8)  D (10.8)  F (8.7) | R (36.2)  L (13.0)  D (11.8)  S (10.7)  A (8.3) | R (33.5)  D (14.6)  L (13.1)  A (8.3)  N (7.3) |
| 45-64 | R (24.9)  D (17.3)  L (15.6)  A (9.3)  S, U (6.8) | L (18.1)  A (15.6)  D (15.0)  R (11.9)  S (10.0) | S (18.3)  L (17.9)  A (13.0)  R (12.2)  K (9.5) | L (21.6)  S (18.5)  A (15.6)  R (8.5)  F (7.4) | L (15.0)  S (14.7)  A (14.1)  D (10.8)  R (10.8) | L (27.5)  S (14.4)  D (10.8)  R (9.3)  K (8.0) | R (40.2)  L (12.2)  S (11.8)  D (8.5)  A/F (5.3) | R (24.0)  L (17.1)  D (14.7)  A (10.9)  P (10.1) |
| 65 and higher | R (21.5)  A (16.1)  D (14.9)  K (13.2)  L (13.2) | A (17.3)  R (17.3)  U (12.8)  L (12.2)  K (10.9) | K (24.7)  A (14.1)  R (11.3)  S (11.0)  L (10.1) | A (23.6)  L (18.4)  R (10.3)  S (9.7)  K, D (9.4) | L (15.7)  A (15.2)  R (12.3)  K (10.2)  D (10.0) | K (20.9)  L (18.3)  R (13.2)  D (12.7)  S (9.8) | R (37.7)  L (14.1)  S (13.6)  D (8.9)  F/P (5.8) | A (17.9)  K (16.7)  R (15.7)  L (14.5)  D (11.1) |
| *A = General and unspecified; D = Digestive; F = Eye; H = Ear; K = Cardiovascular; L = Musculoskeletal; N =Neurologic; R = Respiratory; S = Skin.*  **Switzerland reported 11 contacts in the first age group.* | | | | | | | | |

| **Table S3.** Top 10 diagnosis ICPC (code and %) per country | | | | | | | |
| --- | --- | --- | --- | --- | --- | --- | --- |
| **Countries** | | | | | | | |
| **Belgium**  **(N=1368)** | **Denmark**  **(N=974)** | **Germany**  **(N=1076)** | **Netherlands**  **(N=2048)** | **Norway**  **(N=3000)** | **Slovenia**  **(N=2637)** | **Spain**  **(N=1402)** | **Switzerland**  **(N=649)** |
| R74 (13.0) | A05 (4.9) | S12 (6.3) | L81 (11.4) | A99 (5.6) | L03 (5.6) | R74 (29.9) | R80 (6.4) |
| D73 (12.2) | A03 (2.2) | R74 (4.5) | S18 (8.1) | S18 (4.1) | S18 (5.6) | R76 (4.9) | L84 (5.9) |
| R78 (4.3) | L17 (2.2) | R76 (4.0) | A03 (2.8) | U71 (3.6) | R74 (5.5) | S18 (4.0) | D73 (4.8) |
| A77 (3.8) | R21 (2.2) | A78 (3.8) | D01 (2.6) | R74 (3.2) | L81 (3.7) | S88 (4.0) | R74 (4.8) |
| H71 (3.8) | R81 (2.2) | U71 (3.7) | R74 (2.4) | A03 (2.6) | D01 (2.9) | H72 (3.4) | U71 (2.9) |
| D87 (3.2) | D02 (2.1) | K86 (2.8) | U71 (2.3) | D01 (2.6) | K86 (2.8) | F70 (3.4) | K86 (2.6) |
| R76 (3.2) | H01 (2.1) | R78 (2.6) | A29 (1.8) | L81 (2.0) | U71 (2.7) | L03 (3.2) | A96 (2.5) |
| S18 (2.9) | U71 (2.1) | F70 (2.4) | S76 (1.7) | R05 (1.8) | L86 (2.4) | L99 (3.1) | H82 (2.5) |
| U71 (2.8) | S18 (1.8) | T90 (2.2) | A80 (1.6) | L17 (1.5) | F76 (2.4) | P74 (3.1) | R78 (2.5) |
| A99/R75 (1.8) | S12 (1.8) | H71 (2.1) | D73 (1.4) | F70 (1.3) | R76 (2.3) | R78 (2.9) | A28 (2.3) |
| *A03 Fever; A05 Feeling ill; A28 Limited function/disability NOS; A29 General symptom/complaint other; A77 Viral disease other/ NOS; A78 Infectious disease other/NOS; A80 Trauma/injury NOS; A96 Death; A97 No disease; A99 General disease NOS; D01 Abdominal pain/cramps general; D02 Abdominal pain epigastric; D73 Gastroenteritis presumed infection; D87 Stomach function disorder; F70 Conjunctivitis infectious; F76 Foreign body in eye; H01 Ear pain/earache; H70 Otitis externa; H71 Acute otitis media/myringitis; H82 Vertiginous syndrome; K86 Hypertension uncomplicated; L03 Low back symptom/complaint; L17 Foot/toe symptom/complaint; L81 Injury musculoskeletal NOS; L84 Back syndrome w/o radiating pain; L86 Back syndrome with radiating pain; L99 Musculoskeletal disease, other; P74 Anxiety disorder/anxiety state; R05 Cough; R21 Throat symptom/complaint; R74 Upper respiratory infection acute; R75 Sinusitis acute/chronic R76 Tonsillitis acuta; R78 Acute bronchitis/bronchiolitis; R80 Influenza; R81 Pneumonia; S12 Insect bite/sting; S16 Bruise/contusion; S18 Laceration/cut; S88 Dermatitis contact/ allergic; S76 Skin infection other; S98 Urticaria; T90 Diabetes non-insuline dependent; U71 Cystitis/ urinary infection other* | | | | | | | |
